# Utility of shock index for predicting severity of postpartum haemorrhage: A systematic review and meta-analysis

**DOI:** 10.12669/pjms.41.7.12276

**Published:** 2025-07

**Authors:** Yiyu Pan, Jielan Ding, Juan Feng, Zhengbin Pan

**Affiliations:** 1 Yiyu Pan, Department of Anesthesia, Shaoxing Maternity and Child Health Care Hospital, Shaoxing, Zhejiang Province 312000, P.R. China; 2 Jielan Ding, Department of Anesthesia, Shaoxing Maternity and Child Health Care Hospital, Shaoxing, Zhejiang Province 312000, P.R. China; 3 Juan Feng, Department of Anesthesia, Shaoxing Maternity and Child Health Care Hospital, Shaoxing, Zhejiang Province 312000, P.R. China; 4 Zhengbin Pan, Department of Anesthesia, Shaoxing Maternity and Child Health Care Hospital, Shaoxing, Zhejiang Province 312000, P.R. China

**Keywords:** Diagnostic Accuracy, Meta-Analysis, Postpartum hemorrhage, Shock Index

## Abstract

**Background and Objective:**

Postpartum haemorrhage (PPH) is a leading cause of maternal mortality globally. Shock index (SI), a simple measure of heart rate to systolic blood pressure ratio, has been proposed as a tool to predict severe PPH, but its efficacy varies across different settings. This study aims to assess diagnostic accuracy of SI for severe PPH.

**Methods:**

Comprehensive database searches of EMBASE, CINAHL, PubMed, and MEDLINE Cochrane library was done for studies reporting data on SI in patients with PPH from inception of each database until March 2024. Data on sensitivity, specificity, likelihood ratios, diagnostic odds ratio (OR), and area under the receiver operating characteristic curve (AUC) were extracted. Subgroup analyses were performed based on the income group of the countries and the criteria used to define severe PPH.

**Results:**

Twenty studies were included. Pooled sensitivity and specificity of SI for predicting severe PPH were 76% (95% confidence interval (CI): 67%-83%) and 78% (95% CI: 68%-85%), respectively; diagnostic OR was 11 (95% CI: 5-24), and the AUC was 0.84 (95% CI: 0.80-0.87). Subgroup analysis revealed higher diagnostic accuracy of SI in lower/upper middle-income countries compared to high-income countries. Studies using blood transfusion as a criterion for severe PPH showed higher predictive sensitivity and specificity of the shock compared to studies using quantitative blood loss.

**Conclusion:**

Shock index demonstrates moderate effectiveness in predicting severe PPH and can be a valuable tool in emergency obstetric care, particularly when used as part of a multi-parameter diagnostic approach. Future research should focus on refining its use across different clinical settings.

## INTRODUCTION

Postpartum haemorrhage (PPH) remains a leading cause of maternal morbidity and mortality globally, despite advances in obstetric care.^1^ PPH is defined as blood loss exceeding 500 mL after vaginal birth or 1000 mL following caesarean section, and is associated with significant risks, often necessitating rapid intervention to prevent severe outcomes, including maternal death. The World Health Organization (WHO) identifies PPH as a major global health challenge, with the highest incidence in low-resource settings where healthcare infrastructure may be lacking.^2^ In South-East Asia, PPH incidence ranges from 1.5% to 22.0% of deliveries, especially in Pakistan where PPH accounts for nearly 25% of maternal deaths and results in over 150 000 complications each year.^3^

Management of PPH requires prompt diagnosis and timely therapeutic intervention to mitigate the risk of severe complications.^1^ However, traditional clinical markers, such as the amount of blood loss, are often subjective and prone to underestimation, leading to delays in the diagnosis and treatment of PPH.^4^ Therefore, there is a growing interest in identifying reliable, objective, and easily measurable indicators that can predict the severity of PPH and guide immediate clinical decisions. Shock index (SI) is a simple clinical parameter calculated as the ratio of heart rate to systolic blood pressure.^5^ Recently, it has emerged as a potential predictive tool in PPH.^6^ SI was originally utilized in emergency medicine to assess the severity of haemorrhagic shock and trauma outcomes, and offers a quantitative measure that may more accurately reflect the hemodynamic status of patients compared to traditional method of monitoring vital signs alone.^7^ The appeal of the SI lies in its simplicity, and cost-effectiveness. Moreover, SI may be calculated very quickly, making it potentially invaluable in both high- and low-resource settings.^8^ Several retrospective and prospective studies^9^-^12^ have evaluated SI for predicting PPH severity. Most investigations report that elevated SI on admission is associated with increased risk of transfusion and invasive interventions, although optimal thresholds vary across cohorts. These findings support SI as a useful early marker of severe PPH in diverse clinical settings.

Despite its use in other areas of medicine, value of SI in obstetrics, particularly in the context of PPH, is less clear. Recent studies have suggested that elevated SI could serve as an early marker of significant PPH, potentially enabling timely intervention and tailored management strategies.^6^,^9^-^12^ However, there is still no consensus on the effectiveness and predictive value of SI in patients with PPH.^9^-^12^ This review aimed to explore the utility of SI in predicting the severity of PPH.

## METHODS

Our review encompassed randomized controlled trials (RCTs), as well as observational studies (both prospective and retrospective) that involved patients who experienced PPH following childbirth. Only full-text articles were considered for inclusion, excluding publications that were only available as abstracts or unpublished data. The review included studies that assessed SI in PPH patients, and compared outcomes of patients with various SI values at the onset of haemorrhage. Our review focused on studies providing the data on the severity of PPH, as defined based on any one of the following characteristics: need for blood transfusion, blood loss > 500 mL in normal vaginal delivery or > 1000 mL in caesarean section.

### Search strategy:

A comprehensive electronic search strategy was implemented to gather data from various databases such as EMBASE, CINAHL, PubMed, MEDLINE Cochrane library. The search aimed to collect studies on the utility of SI for predicting the severity of PPH. Specific search terms related to our research focus, including “Shock Index,” “Postpartum Haemorrhage,” “Maternal Health,” “Hemodynamic Stability,” “Obstetric Haemorrhage,” “Blood Loss in Childbirth,” and “Maternal Morbidity,” were used in different combinations across the selected databases. This detailed search was carried out from the start of each database’s records until March 2024, with no language restrictions to ensure the inclusion of international studies, (Supplementary Material).

## SUPPLEMENTARY MATERIAL

**Search Strategy:**

**PubMed:**

#1 “Postpartum Hemorrhage”[Mesh]

OR “postpartum hemorrhage”[tiab]

OR “postpartum haemorrhage”[tiab]

OR “post partum hemorrhage”[tiab]

OR “puerperal hemorrhage”[tiab]

OR “puerperal haemorrhage”[tiab]

OR “obstetric hemorrhage”[tiab]

OR “maternal hemorrhage”[tiab]

OR “maternal haemorrhage”[tiab]

OR (“uterine atony”[tiab] AND (“hemorrhage”[tiab] OR “bleeding”[tiab]))

#2 “Shock Index”[Mesh]

OR “shock index”[tiab]

OR SI[tiab]

OR “S I”[tiab]

OR (heart rate[tiab] AND (“systolic blood pressure”[tiab]

OR “systolic BP”[tiab] OR SBP[tiab])

AND (ratio[tiab] OR quotient[tiab] OR index[tiab]))

OR “HR/SBP”[tiab]

OR “HR:SBP”[tiab]

OR “heart rate/systolic blood pressure ratio”[tiab]

OR “heart rate over systolic blood pressure”[tiab]

#3 #1 AND #2

#4 Limits: Humans;

**EMBASE:**

1. ‘postpartum hemorrhage’/exp

OR ‘postpartum hemorrhage’:ti,ab

OR ‘postpartum haemorrhage’:ti,ab

OR ‘puerperal hemorrhage’:ti,ab

OR ‘puerperal haemorrhage’:ti,ab

OR ‘obstetric hemorrhage’:ti,ab

OR ‘maternal hemorrhage’:ti,ab

OR (uterine atony:ti,ab AND (hemorrhage:ti,ab OR bleeding:ti,ab))

2. ‘shock index’/exp

OR ‘shock index’:ti,ab

OR SI:ti,ab

OR (heart rate:ti,ab AND (’systolic blood pressure’:ti,ab OR SBP:ti,ab) AND (ratio:ti,ab

OR quotient:ti,ab OR index:ti,ab))

OR ‘HR/SBP’:ti,ab

OR ‘HR:SBP’:ti,ab

OR ‘heart rate/systolic blood pressure ratio’:ti,ab

3. 1 AND 2

4. Limit to human;

**CINAHL**:

( MH “Postpartum Hemorrhage+”

OR TI “postpartum hemorrhage”

OR TI “postpartum haemorrhage”

OR TI “post partum hemorrhage”

OR TI “puerperal hemorrhage”

OR TI “puerperal haemorrhage”

OR TI “obstetric hemorrhage”

OR TI “maternal hemorrhage”

OR TI “uterine atony hemorrhage”

OR AB “uterine atony bleeding” )

AND

( MH “Shock Index”

OR TI “shock index”

OR TI SI

OR TI “heart rate/systolic blood pressure ratio”

OR TI “HR/SBP”

OR AB “heart rate to systolic blood pressure quotient” )

Limits: Peer-reviewed; Human

**MEDLINE (via Ovid):**

1. exp Postpartum Hemorrhage/

OR postpartum hemorrhage.tw.

OR postpartum haemorrhage.tw.

OR puerperal hemorrhage.tw.

OR obstetric hemorrhage.tw.

OR maternal hemorrhage.tw.

OR (uterine atony.tw. AND (hemorrhage.tw. OR bleeding.tw.))

2. exp Shock Index/

OR shock index.tw.

OR SI.tw.

OR (heart rate.tw. AND (systolic blood pressure.tw. OR SBP.tw.) AND (ratio.tw. OR

quotient.tw. OR index.tw.))

OR (HR/SBP.tw. OR HR:SBP.tw.)

3. 1 AND 2

4. Limit to humans;

**Cochrane CENTRAL**:

#1 MeSH descriptor: [Postpartum Hemorrhage] explode all trees

OR postpartum hemorrhage:ti,ab,kw

OR postpartum haemorrhage:ti,ab,kw

OR puerperal hemorrhage:ti,ab,kw

OR obstetric hemorrhage:ti,ab,kw

OR maternal hemorrhage:ti,ab,kw

OR uterine atony hemorrhage:ti,ab,kw

#2 MeSH descriptor: [Shock Index] explode all trees

OR shock index:ti,ab,kw

OR SI:ti,ab,kw

OR HR/SBP:ti,ab,kw

OR (heart rate:ti,ab,kw AND (systolic blood pressure:ti,ab,kw) AND (ratio:ti,ab,kw OR

quotient:ti,ab,kw))

#3 #1 AND #2

#4 Apply filters as needed (e.g., trials, diagnostic accuracy).

Final search was conducted on 31 March 2024. No language restrictions were applied. Titles and abstracts of non-English articles were screened using machine translation (e.g., Google Translate). Full texts judged potentially eligible were then translated by bilingual investigators or via professional translation to ensure accurate data extraction.

### Study selection procedure:

**Fig.1:**
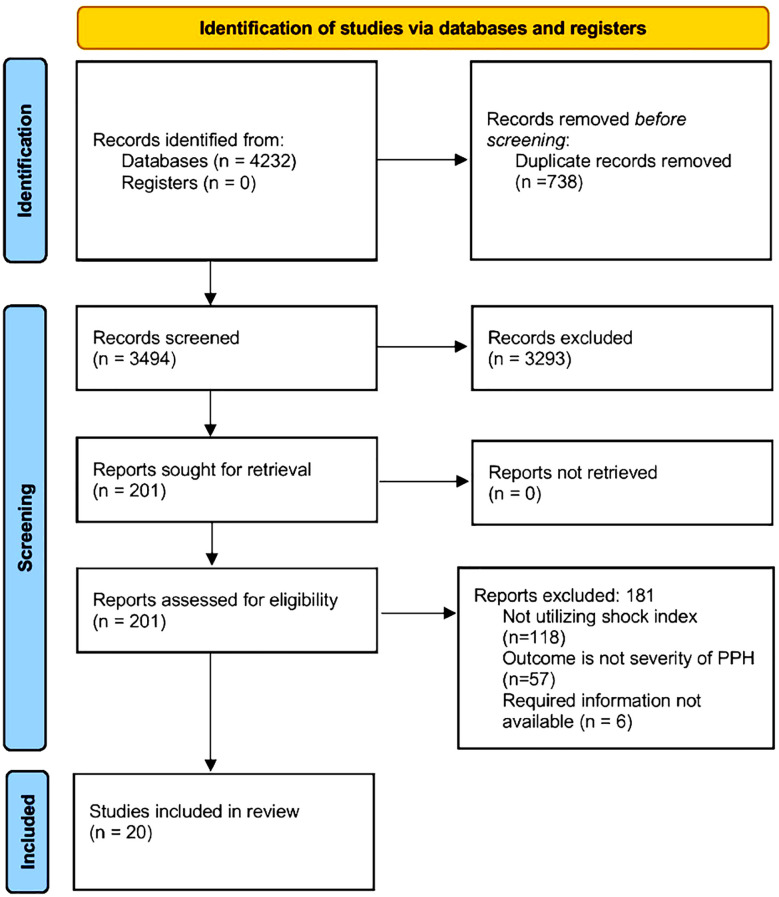
Search strategy flowchart.

All records retrieved from database and grey-literature searches were imported into EndNote X9 (Clarivate) and deduplicated. Duplicates were first identified and removed automatically using matching fields (title, author, year), and then the remaining records were manually checked to eliminate any residual duplicates prior to title/abstract screening. Study selection process was done by the two independent reviewers (YP and JD). Each reviewer first performed individual searches, subsequently screening titles and abstracts of the retrieved studies to identify studies eligible for inclusion. Those studies underwent a thorough full-text review. Both reviewers independently assessed abstracts and full texts, carefully verifying each study against the established inclusion criteria. Discrepancies were resolved by discussion, and if no consensus was reached third senior reviewer (ZP) adjudicated. Inter-rater agreement was quantified using Cohen’s κ statistic (which was estimated to be 0.91) to ensure consistency in study selection. The entire selection process is summarized in a PRISMA flow diagram (Fig.1).

### Data collection:

Data extraction process was directed by the two independent investigators (YP and JD). Following essential data elements were methodically collected from included studies: date of data extraction, study title, and details of the authors, study design, details regarding study participants, and context in which the research was conducted, initial number of participants in each group, baseline and final outcome measures, along with the inclusion and exclusion criteria used for participants, detailed descriptions of the patient groups analysed based on their SI scores at the onset of postpartum haemorrhage, and the follow-up period over which the participants were monitored.

### Risk of bias assessment:

The assessment of study quality was conducted by two independent reviewers (JD & JF) using the Newcastle-Ottawa Scale (NOS), which is designed for the evaluation of non-randomized, observational studies.^13^ This scale assesses studies across three main categories: the selection of the study groups, how comparable the groups are, and the determination of the exposure or outcome of interest, depending on whether the study is a case-control or cohort study. The scale allocates up to nine stars across three domains (selection, comparability, and exposure/outcome). Studies scoring 7–9 stars were classified as low risk of bias, those with 4–6 stars as moderate risk, and those with 0–3 stars as high risk.

### Statistical analysis:

Statistical analysis was done using STATA software, version 14.2. For outcomes presented as dichotomous data, results were reported as odds ratios (OR) along with their 95% confidence intervals (CI). Random-effects model was applied using inverse variance method.^14^ Heterogeneity among the studies was assessed by several methods: examining the overlap of confidence intervals in forest plots, conducting chi-square tests, and calculating I^2^ statistic.^14^ I^2^ statistic more than 75% indicates presence of high inter-study heterogeneity.

Sensitivity and specificity of SI was pooled using bivariate meta-analysis approach. Additionally, positive and negative likelihood ratios (LRP and LRN) and diagnostic odds ratio (DOR) were calculated to determine the utility of SI. The results were displayed using forest plots (pooled specificity and sensitivity) and LR scattergrams (for LRP and LRN). We reported summary of predictive accuracy through “summary receiver operator characteristic curve” (sROC). Heterogeneity between studies was evaluated using a chi-square test and the I^2^ statistic, with the findings visually represented in bivariate box plot. Publication bias was assessed using Deek’s test. We conducted prespecified subgroup analyses by country income level and by severe PPH definition. Countries were stratified according to the 2023 World Bank income groups (low, lower-middle, upper-middle, and high income). Severe PPH was defined using WHO criteria as cumulative blood loss ≥1 000 mL within 24 hours postpartum.

## RESULTS

Four thousand two hundred thirty-two (4232) records were identified by the initial literature search. Of them, 738 duplicates were removed. Of the remaining, 3,494 studies, 201 were retrieved and assessed for eligibility. Finally, 20 studies were included in the review (Fig.1).^6^,^9^-^12^,^15^-^29^

### Characteristics of the included studies:

All 20 studies examined diagnostic accuracy of SI for severe PPH. Studies varied in design, ranging from prospective cohorts to retrospective reviews, and included a sample size totalling over 30,000 participants from middle to high-income countries. Most studies (14 out of 20 studies) defined severe PPH as the need for blood transfusion, with shock index cut-offs ranging from 0.7 to 1.3. Risk of bias was mixed, with eight studies assessed as low risk, seven as moderate, and five as high risk. Mean participant ages, when reported, generally ranged in the early to mid-30s (Supplementary Table-I).

**Supplementary Table-I:** Included studies core information.

Author, year	Country	study region	Study design	Participant details	Overall sample size	Diagnostic criteria for severe PPH	Cut-off of shock index	Mean age of the participants	Risk of bias assessment (NOS score)
Agarwal 2021	India	Middle income countries	Prospective cohort study	Patients who delivered after 28 weeks with visual blood loss greater than 500 ml in normal vaginal delivery and greater than 1000 ml during LSCS	100	Need for blood transfusion	1.3	27.04 + 2.99	Moderate (5)
Agarwal 2023	India	Middle income countries	Retrospective cohort study	Patients having PPH at the department of obstetrics and gynaecology at a tertiary care hospital in northern india.	105	Need for blood transfusion	1.1	26.47 (21-38)	Moderate (6)
Attali 2022	Israel	High income countries	Retrospective cohort study	Patients with post-partum hemorrhage (PPH), defined clinically as an estimated excessive bleeding (more than 500 ml following a vaginal delivery, and more than 1000 ml following a cesarean delivery) requiring uterotonic drugs and fluid resuscitation	94	PPH was defined as estimated excessive blood loss (of more than 500 ml following vaginal delivery and 1000 ml following a caesarean delivery) requiring at least one uterotonic drug and fluid resuscitation.	NR	Non-transfused group = 33.7 (4.9) Transfused group = 32.0 (6.3)	High (2)
Borovac-pinheiro 2018	Brazil	Middle income countries	Retrospective case–control study	Women who required blood transfusion due to PPH (study group) control women who delivered in the same period and required no blood transfusion.	180	The current criterion for PPH diagnosis is an estimated blood loss of more than 500 ml.	0.83	NR	High (1)
Butt 2022	Pakistan	Middle income countries	Retrospective observational study	Women who were referred with the diagnosis of pph.	197	Need for blood transfusion.	1.15	28.71± 5.33	Moderate (5)
Drew 2021	Canada	High income countries	Prospective observational study	Labouring women (gestational age >37 weeks) undergoing spontaneous or instrumental vaginal delivery	66	Need for blood transfusion	0.9 (for >1000 ml blood loss)	32.1 (4)	Moderate (5)
Era 2015	Japan	High income countries	Retrospective observational study	Patients who had received blood transfusions	80	Requirement of blood transfusion or estimated blood loss of 2000 ml or more; fibrinogen level of 150 mg/dl or less;and hb of 7 g/dl or less.jsog dic score of 8 or more,and si of 1 or more.	1.12	NR	Moderate (6)
Kong 2021	South Korea	High income countries	Retrospective cohort study	Patients with primary PPH who were prospectively integrated into the speed cp program.	278	Primary PPH was defined as a hemorrhage requiring fluid resuscitation or transfusion within the first 24 h of delivery	1	32.70±3.95	High (3)
Kwon 2024	South Korea	High income countries	Retrospective observational study	Patients with primary PPH	612	Need for blood transfusion	1.07	32.0 (30.0-35.0)	Low (7)
Lee 2019	South Korea	High income countries	Retrospective study	Patients with primary PPH	118	Need for blood transfusion	0.7	32.5 ± 5.1	High (2)
Mori 2021	Japan	High income countries	Retrospective case–control study	Patients with hypertensive disorders in pregnancy in a singleton tertiary perinatal medical facility	107	Need for blood transfusion	0.99 (for > 1500 ml blood loss)	32 (19–44)	Moderate (5)
Nathan 2015	UK	High income countries	Retrospective cohort study.	Women with PPH over a 1-year period.	233	Need for blood transfusion	0.9	32.2 (5.9)	Low (8)
Nwafor 2020	Nigeria	Middle income countries	Retrospective cohort study	Women treated for primary postpartum hemorrhage	289	Need for blood transfusion	0.9	26±4.2	Low (8)
Okada 2020	Japan	High income countries	Retrospective cohort study	Patients with PPH	60	NR	1.1	Massive transfusion (mt,19) = 36.0 (32.0–38.0) Non-mt (n = 12) = 32.5 (30.5–36.0)	Moderate (4)
Pacagnella 2022	Brazil	Middle income countries	Prospective cohort study	Women who delivered vaginally	270	Need for blood transfusion	0.785 for > 1000 ml in 2 hours	24.67 ± 6.19	Low (9)
Sert 2021	Turkey	Middle income countries	Prospective study	Female patients with more than 500 ml of bleeding after vaginal delivery	201	Need for blood transfusion.	0.81 (for > 1000 ml blood loss)	Severe PPH (n = 43) = 26.9 ± 5.9 Non-severe PPH (n = 158) = 25.5 ± 4.9	Low (7)
Sohn 2013	South Korea	High income countries	Retrospective cohort study	Primary PPH patients	126	Primary PPH was defined as blood loss of 500 ml or more that occurs within 24 h after birth	1.3	Massive transfusion (mt,26) = 31.0 (29.8y34.5) Non-mt (n = 100) = 31.0 (29.0y34.0)	Low (8)
Sohn 2018	South Korea	High income countries	Retrospective cohort study	Patients with primary postpartum haemorrhage	390	Primary PPH was defined as haemorrhage requiring transfusion or fluid resuscitation within the first 24 h of delivery.	1	Massive transfusion (mt,101) = 32.0 (30.0, 35.5) Non-medical treatment (n = 201) = 32.0 (29.0, 34.0)	High (2)
Tanacan 2020	Turkey	Middle income countries	Retrospective case-control study	Pregnant women with singleton pregnancies	260	Need for blood transfusion	0.91	Cases= 30.23 ± 5.38 Control= 30.10 ± 5.60	Low (8)
Ushida 2021	Japan	High income countries	Retrospective study	Women who delivered vaginally	30,820	Need for blood transfusion	0.83 (for >1500 ml blood loss)	31 (28–34)	Low (7)

h – hours; mt – Massive transfusion; NOS – Newcastle Ottawa Scale; PPH – Postpartum hemorrhage; y-years.

### Diagnostic accuracy of shock index for severe form of PPH:

**Fig.2:**
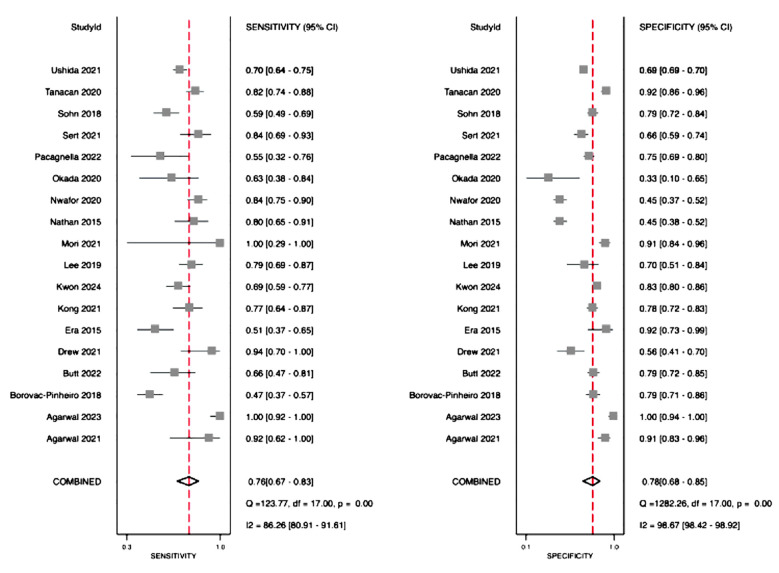
Forest plot showing the pooled sensitivity and specificity of shock index for predicting severe PPH.

**Fig.3:**
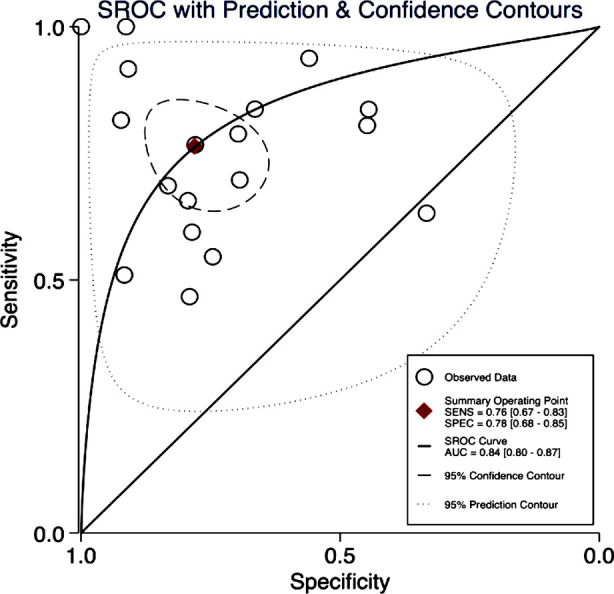
Summary ROC curve for shock index to predict severe PPH.

Pooled sensitivity and specificity of SI for predicting severe PPH was 76% (95%CI: 67%-83%) and 78% (95%CI: 68%-85%), respectively (Fig.2). LRP was 3.5 (95%CI: 2.2 to 5.4), LRN was 0.30 (95%CI: 0.21 to 0.45) and diagnostic odds ratio was 11 (95% CI: 5 to 24). The AUC value was 0.84 (95%CI: 0.80 to 0.87) (Fig.3). The LR scattergram showed that the point estimate was in right lower quadrant indicating that SI cannot be used for confirmation or exclusion of severe PPH.

**Fig.4:**
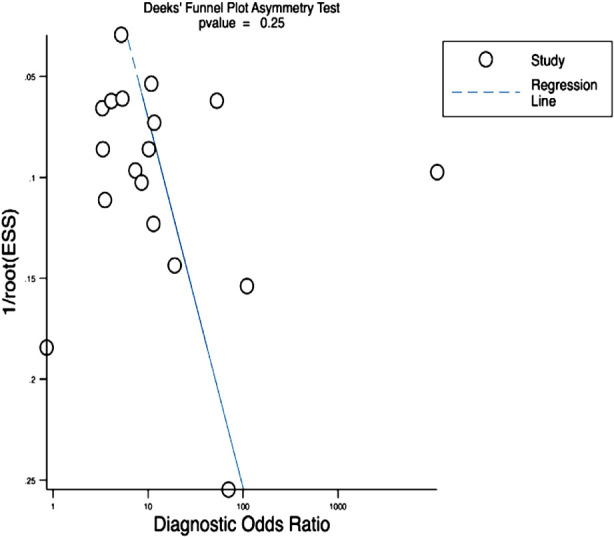
Funnel plot applying Deek’s test.

**Fig.5:**
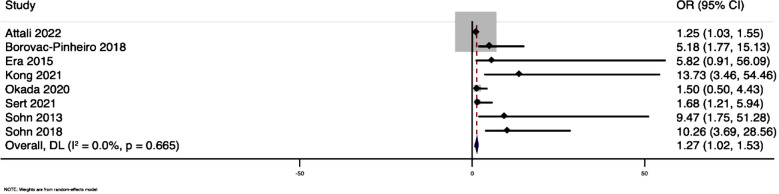
Forest plot showing the risk of severe PPH based on the values of shock index.

Subgroup analysis based on income group region of countries showed that studies done in lower/upper middle-income group countries exhibited higher pooled sensitivity (82%; 95% CI: 62%-93%) and specificity (84%; 95%CI: 67%-94%) when compared to high-income countries (sensitivity=70%; 63%-77%; specificity=74%; 95%CI: 62%-83%). Analysis based on the outcome definition of severe PPH showed that studies that used a need for blood transfusion as criteria for diagnosing severe PPH had higher sensitivity (76%; 95%CI: 64%-84%) and specificity (80%; 95%CI: 66%-89%) when compared to studies that used the amount of bleed (>1000 mL or >1500 mL) as an outcome criteria (sensitivity=69%; 95%CI: 53%-81%; specificity=72%; 95%CI: 60%-82%). Bivariate box plot showed significant heterogeneity, which was further confirmed by the I-squared value of 97%. Deek’s funnel plot asymmetry test (Fig.4) showed that there was no publication bias (p-value of 0.25). We also assessed the risk of severe form of PPH as dichotomous outcome. Pooled OR for the utility of SI for predicting risk of severe PPH was 1.27 (95%CI: 1.01-1.53; I-squared statistic=0%) (Fig.5). Sensitivity analysis excluding studies with moderate or high risk of bias did not show any significant variation in pooled sensitivity and specificity.

## DISCUSSION

This review evaluated diagnostic accuracy of SI in predicting severe PPH. The pooled results indicated that SI has 76% sensitivity and 78% specificity for detecting severe PPH. Our subgroup analysis revealed significant variability in diagnostic accuracy based on the income group of the countries where studies were conducted, with higher sensitivity and specificity observed in lower/upper middle-income countries compared to high-income countries. This variation could be influenced by differences in clinical practices, healthcare settings, and population health dynamics. Another important finding from the subgroup analysis is that the definition of severe PPH impacts the diagnostic accuracy of SI. Studies that used the need for blood transfusion as a criterion showed better diagnostic performance compared to those defining severe PPH by the amount of blood loss.

Our findings are in line with previous research indicating that SI can serve as a useful early warning indicator for severe PPH.^30^ For instance, studies have shown that higher SI is associated with increased morbidity and mortality in haemorrhaging patients. However, as opposed to some studies that suggest that SI may serve as a highly reliable standalone predictor, our findings imply that SI alone is not enough for making informed clinical decisions regarding PPH management.^30^ In contrast to studies advocating for universal cut-offs, our analysis underscores the impact of local context on the diagnostic accuracy of SI, particularly highlighting differences between income groups.^31^ This is an area that has not been extensively covered in existing literature. Our results suggest that global guidelines might need to be adjusted to better fit regional healthcare capacities and practices.

Our results also add granularity to the literature by demonstrating that the operational definition of severe PPH (e.g., blood loss vs. transfusion need) can significantly affect the performance of diagnostic indicators like SI. This finding is crucial as it suggests that SI may perform differently under varying clinical criteria and conditions, which is not widely reported in existing studies.^30^ Moderate diagnostic accuracy of SI in predicting severe PPH can be attributed to several factors. SI is calculated as the ratio of heart rate to systolic blood pressure, and provides a dynamic assessment of hemodynamic instability, which is a key feature of severe PPH.^32^ Hemodynamic responses to haemorrhage can vary widely among patients, and may be influenced by factors such as age, comorbidities, and baseline cardiovascular health.^33^ These variations can impact sensitivity and specificity of SI in detecting severe PPH. Clinical presentation of severe PPH may differ based on factors such as the timing of assessment, presence of confounding variables (e.g., concurrent medical conditions), and the availability of resources for timely intervention.^34^

Additionally, differences in the demographics and obstetric profiles of study populations, including gestational age, parity, and obstetric history, can affect hemodynamic response to PPH and, consequently, diagnostic performance of SI.^35^ Variations in healthcare infrastructure, clinical protocols, and access to interventions across different settings may impact the interpretation and application of SI,^36^ while differences in clinical practices, including thresholds for intervention and transfusion triggers, can influence its diagnostic utility.

### Limitations:

However, our study has some limitations. We identified significant heterogeneity across studies, which may have influenced the pooled estimates of diagnostic accuracy. Variability in the quality and methodology of the included studies, including differences in diagnostic criteria and reference standards, may have introduced bias and confounded the results. The findings of the review may be limited by the geographical and demographic characteristics of the included studies, potentially limiting the generalizability of the results to other populations and settings. Nonetheless, we have performed a robust and comprehensive review following PRISMA-DTA guidelines.^37^

Our findings have several important implications for clinical practice in the management of PPH. SI, while not definitive on its own, can be a valuable component of a multi-parameter approach to early identification and management of severe PPH. It offers a quick, non-invasive, and cost-effective method to assess hemodynamic status, which is crucial in time-sensitive situations like PPH where rapid decision-making can significantly impact outcomes. Given its moderate sensitivity and specificity, SI should be used in conjunction with other clinical indicators and assessments. This integrated approach can enhance diagnostic accuracy and facilitate timely interventions. Healthcare settings, especially in lower and middle-income countries where resources may be limited, should consider integrating SI into standard operating protocols for managing PPH. This tool can aid in stratifying patients based on risk and prioritizing care for those at higher risk of severe outcomes.

While this review provides important insights, several areas require further investigation to enhance the utility of the shock index in clinical settings. There is a need for prospective studies that evaluate SI in real-time clinical settings to validate its utility and determine optimal threshold values specific to PPH. Research into the physiological mechanisms that influence SI during PPH could provide deeper insights into its variability and potential limitations. Studies assessing how interventions based on SI thresholds affect clinical outcomes in patients with PPH could help establish evidence-based protocols.

## CONCLUSION

This review has demonstrated that SI is a moderately-effective tool for predicting severe PPH, offering rapid assessment of hemodynamic instability that can be critical in emergency obstetric care. Integration of SI into clinical protocols should be informed by local healthcare capabilities and combined with other clinical assessments to ensure the best outcomes for PPH patients.

***PROSPERO registration number:*** CRD42024531656

### Authors’ contributions:

**YP:** Literature search, Study design and manuscript writing.

**JD, JF and ZP:** Data collection, data analysis and interpretation. Critical review.

**YP:** Critical analysis, manuscript revision and validation and is responsible for the integrity of the study.

All authors have read and approved the final manuscript.

## References

[1] Rani PR, Begum J (2017). Recent Advances in the Management of Major Postpartum Haemorrhage - A Review. J Clin Diagn Res.

[2] WHO recommendations:uterotonics for the prevention of postpartum haemorrhage.

[3] National Guidelines for the Management of Post-Partum Haemorrhage (PPH) for Pakistan.

[4] Atukunda EC, Mugyenyi GR, Obua C, Atuhumuza EB, Musinguzi N, Tornes YF (2016). Measuring Post-Partum Haemorrhage in Low-Resource Settings:The Diagnostic Validity of Weighed Blood Loss versus Quantitative Changes in Hemoglobin. PLoS One.

[5] Allgöwer M, Burri C (1967). Shock index.

[6] Borovac-Pinheiro A, Ribeiro FM, Morais SS, Pacagnella RC (2019). Shock index and heart rate standard reference values in the immediate postpartum period:A cohort study. PLoS One.

[7] Koch E, Lovett S, Nghiem T, Riggs RA, Rech MA (2019). Shock index in the emergency department:utility and limitations. Open Access Emerg Med.

[8] Cheng TH, Sie YD, Hsu KH, Goh ZNL, Chien CY, Chen HY (2020). Shock Index:A Simple and Effective Clinical Adjunct in Predicting 60-Day Mortality in Advanced Cancer Patients at the Emergency Department. Int J Environ Res Public Health.

[9] Kwon H, Sohn CH, Kim SM, Kim YJ, Ryoo SM, Ahn S (2024). Comparison of Modified Shock Index and Shock Index for Predicting Massive Transfusion in Women with Primary Postpartum Hemorrhage:A Retrospective Study. Med Sci Monit.

[10] Mori H, Shibata E, Kuwazuru T, Uchimura T, Kondo E, Yoshino K (2021). The utility of shock index and heart rate in the management of postpartum blood loss in pregnant women complicated with hypertensive disorders in pregnancy. J Obstet Gynaecol Res.

[11] Pacagnella RC, Borovac-Pinheiro A, Silveira C, Siani Morais S, Argenton JLP, Souza JP (2022). The golden hour for postpartum hemorrhage:Results from a prospective cohort study. Int J Gynaecol Obstet.

[12] Tanacan A, Fadiloglu E, Unal C, Beksac MS (2020). Importance of shock index in the evaluation of postpartum hemorrhage cases that necessitate blood transfusion. Women Health.

[13] Lo CKL, Mertz D, Loeb M (2014). Newcastle-Ottawa Scale:comparing reviewers'to authors'assessments. BMC Med Res Methodol.

[14] Cumpston M, Li T, Page MJ, Chandler J, Welch VA, Higgins JP (2019). Updated guidance for trusted systematic reviews:a new edition of the Cochrane Handbook for Systematic Reviews of Interventions. Cochrane Database Syst Rev.

[15] Sert ZS (2021). Prognostic capacity of inferior vena cava diameter for severe postpartum hemorrhage. Eur J Obstet Gynecol Reprod Biol.

[16] Nwafor J, Obi V, Onwe B, Ugojis D, Onuchukwu V, Obi C (2019). Comparison of performance of shock index and conventional vital sign parameters for prediction of adverse maternal outcomes following major postpartum hemorrhage in Abakaliki, Southeast Nigeria. Tropical J Obstet Gynae.

[17] Agarwal S, Pandey U, Kumar L (2023). Shock index as a predictor of maternal outcome in postpartum hemorrhage:an experience from a tertiary care centre in Northern India. Int J Med Rev Case Rep.

[18] Kong T, Lee HS, Jeon SY, You JS, Lee JW, Chung HS (2021). Delta neutrophil index and shock index can stratify risk for the requirement for massive transfusion in patients with primary postpartum hemorrhage in the emergency department. PLoS One.

[19] Butt S, Sattar S, Anbreen T (2022). Shock Index as a Predictor of Adverse Maternal Outcome In Postpartum Hemorrhage. J Surgery Pak.

[20] Attali E, Many A, Kern G, Reicher L, Kahana A, Shemer A (2022). Predicting the need for blood transfusion requirement in postpartum hemorrhage. J Matern Fetal Neonatal Med.

[21] Agarwal V, Suri J, Agarwal P, Gupta S, Mishra PK, Mittal P (2021). Shock Index as a Predictor of Maternal Outcome in Postpartum Hemorrhage. J South Asian Federation Obstet Gynaecol.

[22] Nathan HL, El Ayadi A, Hezelgrave NL, Seed P, Butrick E, Miller S (2015). Shock index:an effective predictor of outcome in postpartum haemorrhage?. BJOG.

[23] Sohn CH, Kim YJ, Seo DW, Won HS, Shim JY, Lim KS (2018). Blood lactate concentration and shock index associated with massive transfusion in emergency department patients with primary postpartum haemorrhage. Br J Anaesth.

[24] Drew T, Carvalho JCA, Subramanian C, Yoon EW, Downey K, Thorneloe B (2021). The association of shock index and haemoglobin variation with postpartum haemorrhage after vaginal delivery:a prospective cohort pilot study. Int J Obstet Anesth.

[25] Okada A, Okada Y, Inoue M, Narumiya H, Nakamoto O (2020). Lactate and fibrinogen as good predictors of massive transfusion in postpartum hemorrhage. Acute Med Surg.

[26] Lee SY, Kim HY, Cho GJ, Hong SC, Oh MJ, Kim HJ (2019). Use of the shock index to predict maternal outcomes in women referred for postpartum hemorrhage. Int J Gynaecol Obstet.

[27] Sohn CH, Kim WY, Kim SR, Seo DW, Ryoo SM, Lee YS (2013). An increase in initial shock index is associated with the requirement for massive transfusion in emergency department patients with primary postpartum hemorrhage. Shock.

[28] Ushida T, Kotani T, Imai K, Nakano-Kobayashi T, Nakamura N, Moriyama Y (2021). Shock Index and Postpartum Hemorrhage in Vaginal Deliveries:A Multicenter Retrospective Study. Shock.

[29] Era S, Matsunaga S, Matsumura H, Murayama Y, Takai Y, Seki H (2015). Usefulness of shock indicators for determining the need for blood transfusion after massive obstetric hemorrhage. J Obstet Gynaecol Res.

[30] Makino Y, Miyake K, Okada A, Ikeda Y, Okada Y (2022). Predictive accuracy of the shock index for severe postpartum hemorrhage in high-income countries:A systematic review and meta-analysis. J Obstet Gynaecol Res.

[31] Aleka P, Van Koningsbruggen C, Hendrikse C (2023). The value of shock index, modified shock index and age shock index to predict mortality and hospitalisation in a district level emergency centre. Afr J Emerg Med.

[32] Agaba DC, Lugobe HM, Migisha R, Jjuuko M, Saturday P, Kisombo D (2024). Abnormal obstetric shock index and associated factors among immediate postpartum women following vaginal delivery at a tertiary hospital in southwestern Uganda. BMC Pregnancy Childbirth.

[33] Scully CG, Daluwatte C, Marques NR, Khan M, Salter M, Wolf J (2016). Effect of hemorrhage rate on early hemodynamic responses in conscious sheep. Physiol Rep.

[34] Nyfløt LT, Sandven I, Stray-Pedersen B, Pettersen S, Al-Zirqi I, Rosenberg M (2017). Risk factors for severe postpartum hemorrhage:a case-control study. BMC Pregnancy Childbirth.

[35] Sebghati M, Chandraharan E (2017). An update on the risk factors for and management of obstetric haemorrhage. Womens Health (Lond).

[36] Padkins M, Kashani K, Tabi M, Gajic O, Jentzer JC (2024). Association between the shock index on admission and in-hospital mortality in the cardiac intensive care unit. PLoS One.

[37] Salameh JP, Bossuyt PM, McGrath TA, Thombs BD, Hyde CJ, Macaskill P (2020). Preferred reporting items for systematic review and meta-analysis of diagnostic test accuracy studies (PRISMA-DTA):explanation, elaboration, and checklist. BMJ.

